# Prophylactic Application of CpG Oligonucleotides Augments the Early Host Response and Confers Protection in Acute Melioidosis

**DOI:** 10.1371/journal.pone.0034176

**Published:** 2012-03-20

**Authors:** Barbara M. Judy, Katherine Taylor, Arpaporn Deeraksa, R. Katie Johnston, Janice J. Endsley, Sudhamathi Vijayakumar, Judith F. Aronson, D. Mark Estes, Alfredo G. Torres

**Affiliations:** 1 Department of Pathology, University of Texas Medical Branch, Galveston, Texas, United States of America; 2 Department of Microbiology and Immunology, University of Texas Medical Branch, Galveston, Texas, United States of America; 3 Sealy Center for Vaccine Development, University of Texas Medical Branch, Galveston, Texas, United States of America; 4 Department of Infectious Diseases, College of Veterinary Medicine, University of Georgia, Athens, Georgia, United States of America; University of California Los Angeles, United States of America

## Abstract

Prophylactic administration of CpG oligodeoxynucleotides (CpG ODNs) is known to confer protection against lethal sepsis caused by *Burkholderia pseudomallei* in the mouse model. The mechanisms whereby CpG regulates the innate immune response to provide protection against *B. pseudomallei*, however, are poorly characterized. In the present study, we demonstrate that intranasal treatment of mice with Class C CpG, results in recruitment of inflammatory monocytes and neutrophils to the lung at 48 h post-treatment. Mice infected with *B. pseudomallei* 48 h post-CpG treatment had reduced organ bacterial load and significantly altered cytokine and chemokine profiles concomitant with protection as compared to control animals. CpG administration reduced the robust production of chemokines and pro-inflammatory cytokines in blood, lung and spleen, observed following infection of non-treated animals. Death of control animals coincided with the time of peak cytokine production (day 1–3), while a moderate; sustained cytokine production in CpG-treated animals was associated with survival. In general, CpG treatment resulted in diminished expression of cytokines and chemokines post-infection, though IL-12p40 was released in larger quantities in CpG treated animals. In contrast to CpG-treated animals, the lungs of infected control animals were infiltrated with leukocytes, especially neutrophils, and large numbers of necrotic lesions were observed in lung sections. Therapeutic treatment of *B. pseudomallei*-infected animals with CpG at 24 h post-infection did not impact survival compared to control animals. In summary, protection of CpG-treated animals was associated with recruitment of inflammatory monocytes and neutrophils into the lungs prior to infection. These responses correspond with early control of bacterial growth, a dampened inflammatory cytokine/chemokine response, reduced lung pathology, and greatly increased survival. In contrast, a delay in recruitment of inflammatory cell populations, despite a robust production of pro-inflammatory cytokines, was associated with poorly controlled bacterial growth, severe lung pathology, and death of control animals.

## Introduction

In the absence of a licensed vaccine, *Burkholderia pseudomallei*, the etiologic agent for melioidosis, presents a significant threat as a potential agent for bioterrorism due to its high rate of infectivity if acquired via the respiratory tract, combined with resistance to many commonly used antibiotics [1]. This intracellular Gram negative bacteria is endemic throughout Northern Australia and parts of Southeast Asia, where as much as 20% of septicemia is attributed to melioidosis [1], [2]. Previous studies have shown that immunization with oligodeoxynucleotides containing unmethylated deoxycytosine-deoxyguanosine motifs (type B) can protect mice from low doses of aerosol or intraperitoneally administered *Burkholderia* infection [3], [4], [5]. CpG ODNs have also been administered alone as a pretreatment to effectively protect mice from infection by different bacterial pathogens [6].

CpG ODNs are recognized by Toll-like receptor 9 (TLR9) and are potent inducers of the innate immune system, mimicking the effects of bacterial DNA. Three classes of CpG ODNs that act as TLR9 agonists have been described with different abilities to activate immune system. Class A induce very high levels of IFN-α produced by plasmocytoid dendritic cells, class B induce strong B and NK cell activation and class C combine most efficiently the properties of both the A and B classes [7], [8], [9], [10], and induce preferential development and differentiation of T helper 1 (Th1) cells [10], [11]. The CpG ODNs can potentially be used in broad applications as a vaccine adjuvant, a stand-alone therapy, or in combination with other therapies in cancer, infectious diseases, asthma and allergy [12], [13], [14]. As such, the use of CpG ODNs may be critical in developing an effective immune response to *B. pseudomallei* [2], [9], [11], [15].

CpG type B pre-treatment resulted in elevated serum levels of IL-12p70 and IFN-γ and decreased bacteremia in BALB/c mice challenged i.p. with *B. pseudomallei* [5]. CpG, type B was also protective against aerosol exposure to the related pathogen *B. mallei*, the etiologic agent of glanders [4]. Additionally, CpG administration enhanced digestion of apoptotic neutrophils by macrophage populations, accompanied with an increase in the level of TNF-α [16]. Such a mechanism could limit dissemination and replication of *B. pseudomallei* by promoting antibacterial and phagocytic activity by these innate immune populations to limit bacterial numbers and inflammation. In an acute lung injury murine model, administration of CpG reduced neutrophil mobilization to alveolar spaces as well as the pro-inflammatory cytokine response [17].

Infections of both humans and mice have been reported to have elevated levels of a number of pro-inflammatory cytokines and mediators following *B. pseudomallei* infection [5], [18], [19], [20]. While a cytokine response may assist recovery, as evidenced by the protection of animals administered IFN-γ, the hyper-responsive production of pro-inflammatory cytokines may induce immunopathology [20], [21], [22], [23]. Because the development of the acute form of melioidosis in BALB/c mice seems to be associated with an increase in inflammation [24], the broad analysis of chemokines and cytokines would be a useful platform for investigating protective role of CpG ODN and could further identify candidate cells of innate immune system that contribute to protective or pathological responses.

As this study demonstrates, early activation of innate immunity via a TLR9 agonist (CpG ODN Type C) alters the cytokine profiles and shifts the innate host defense response to *B. pseudomallei* in a protective manner. Activation of the innate immune system prior to infection to reduce the severity of the disease in susceptible populations could provide a window of opportunity for adequate treatment, which in the case of melioidosis could be critical. Our results significantly advance the understanding regarding innate immunity in both protection and immunopathogenesis following *B. pseudomallei* infection, and describe the protective responses due to CpG ODN treatment applicable to development of medical countermeasures for melioidosis.

## Results

### Effects of CpG ODN treatment on survival

**Figure 1:**
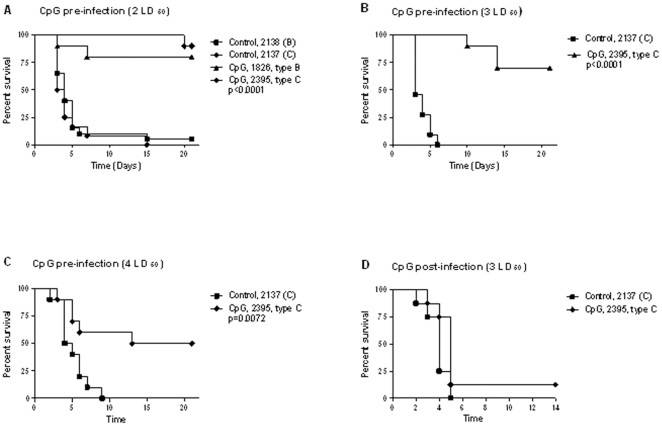
Survival curves of *B. pseudomallei* strain K96243. Percentage of survival of BALB/c mice challenged intranasally with 2 LD_50_ (panel A), 3 LD_50_ (panel B), 4 LD_50_ (panel C) of *B. pseudomallei* strain K96243 (n = 10). CpG ODN 1826 type B or ODN 2395, type C was administrated 48 h prior to infection at 20 µg/mouse in 50 µl of water. Control animals received non-CpG ODN, for type B ODN 2138 and ODN 2137 for type C. The infection of *B. pseudomallei* resulted in 90–100% death in non-treated animals. Survival of CpG treated animals ranged from 80–90% for 2 LD_50_ to 50% for 4 LD_50_. Post-challenge (1 h) administration of CpG ODN type C did not protect animals and both control and CpG treated animals died between days 2 and 5 (panel D). Kaplan-Meier analysis followed by log rank test was used to determine statistical differences.

BALB/c mice are highly susceptible to *B. pseudomallei* infection and provide an excellent model for acute melioidosis. A previous study demonstrates that administration of CpG ODN type B at 48 h before challenge is optimal for protection from *B. pseudomallei* [5]. Therefore, eight-week old female BALB/c mice were given 20 µg of CpG ODN 2137, type C i.n. 48 h prior to intranasal challenge with *B. pseudomallei* K96243 (2–4 LD_50_). For comparison between type C and type B CpG ODN, we treated an additional group of BALB/c mice with type B CpG ODN 2138. We observed that the non-treated control mice became sick within 48 h post-challenge as indicated by non-specific signs such as piloerection and hypoactivity with trembling. As expected, the majority of deaths in the control group occurred within 3 to 6 days post-infection (Figure 1). Our data demonstrated clearly that pretreatment with both CpG ODN type B or C significantly prolonged the survival time in *B. pseudomallei* infected animals. Administration of CpG ODN type B or C 48 h prior to i.n. infection with *B. pseudomallei* (2 LD_50_) resulted in 80% to 100% protection in all experiments (Figure 1A). The studies using higher infectious doses, and for the remainder of our studies, were performed with CpG ODN 2137, type C which has been shown to be optimal for augmenting broader immune responses [10]. A lower degree of protection (70% and 50%) was still observed when higher challenge doses (3LD_50_ or 4 LD_50_) were given to the mice (Figures 1B and C). In all experiments, days 2 and 3 post infection was a consistent turning point for disease progression in susceptible BALB/c mice and suggested that early proper activation of innate immunity can be extremely important in protection against intracellular bacteria in the acute form of disease. In contrast, post-infection with CpG type C treatment was not protective and most animals from both treated and control groups died between days 2 and 5 (Fig. 1D).

### Effects of CpG ODN on bacterial load and the course of infection

To test the effect of CpG ODN 2395 (C-class), we examined the kinetics of bacterial clearance or colonization in BALB/c mice infected i.n. with *B. pseudomallei* K96243 (dose 2LD_50_). We isolated residual bacteria from two animals euthanized at each time point from control and CpG treated group on days 1, 2, 3, 6, and 13 post-infection and at the end of the study. Most untreated control animals died between day 3 to 6 and CFU determination data after day 6 was not available from this control group of animals.

**Figure 2:**
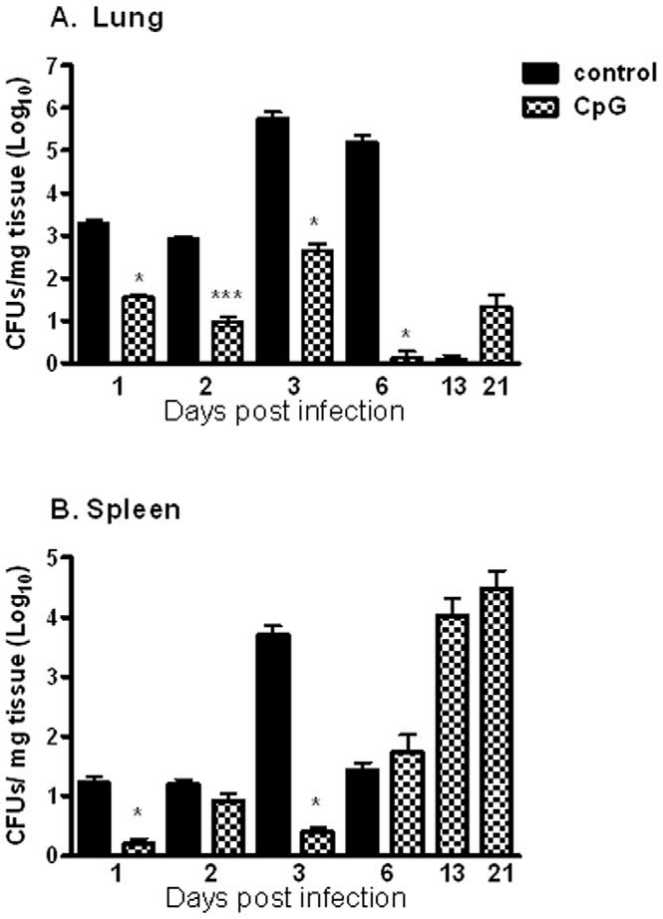
Bacterial recovery from infected organs. Bacterial burden in lungs (A) and spleens (B) were determined in two mice per time point. Mice received CpG ODN, type C 48 h prior challenge with *B. pseudomallei*. Values are expressed as log_10_ CFU per mg of tissue and presented as means ± SEM. Statistical differences were determined using two-tailed Student t test. *p<0.05, ***p<0.001.

Bacteria were detected in lungs and spleens 24 h post infection in both groups of animals (Figure 2). However, animals treated with CpG had reduced bacterial growth in lungs compared to the control group (Figure 2A). On day 1 post-infection, only 36 CFUs were found per mg tissue in the animals treated with CpG and number of bacteria had reached the highest level of 456 CFUs/mg by day 3. By day 6 post-infection, the bacterial load was substantially reduced as compared to day 1 and only few CFUs were detected in lungs per mg of tissue. A low level of bacteria (1.0–22 CFUs/mg) in the lungs of CpG- treated animals was maintained during the remaining course of infection (Figure 2A). Control animals displayed higher levels of bacteria in lungs starting at 24 h post-infection as compared to CpG treated mice. By day 6, the mean number of bacteria found in lungs was 1.62×10^5^ CFUs/mg of tissue, and most of control animals died around that day. The surviving CpG treated animals also controlled bacterial growth in the spleen better than untreated controls during the first three days post-infection (Figure 2B). However, though CpG-treated animals survived and had low organ bacterial burdens at the time that most control animals died, by day 6, bacteria increased in spleen in the CpG treated mice and continued to increase by the end of the study (3 weeks).

### Histopathology

**Figure 3:**
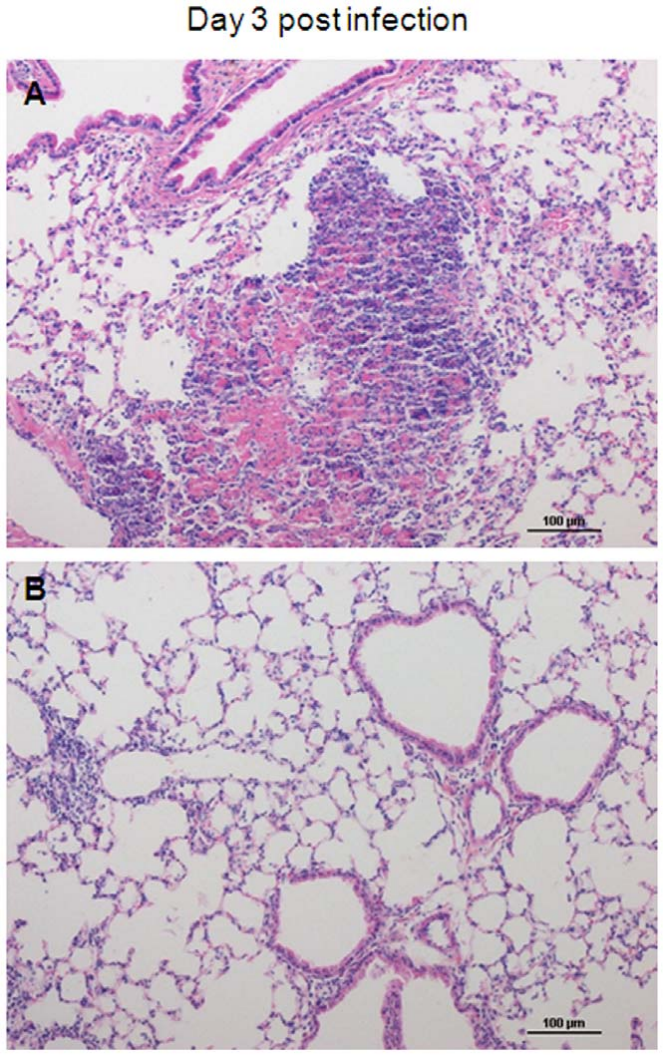
Histological analysis. Representative images from Hematoxylin and Eosin stained mouse lungs for control (A) and CpG treated (B) animals at day 3 post infection. At day 3 post infection, control animals show large nodular, confluent areas of alveolar inflammation with necrosis, whereas inflammation in CpG treated animals is sparse, peribronchial and perivascular in location, and without necrosis.

Lungs from all infected animals at days 1–6 post infection showed multifocal abscess formation dominated by neutrophils with few admixed macrophages. In CpG treated animals, lungs had fewer and smaller lesions than did control animals (Table 1) (Figure 3). Lesion numbers for both groups peaked at day 2–3. At all-time points, control animals had 3–15 times more lesions in lung sections examined than did CpG treated animals. For the CpG treated animals, beginning on day 6 post infection, lesions were difficult to find and relatively small, occupying only 1–2 alveolar spaces. By contrast, in control animals, severe lesions were still seen at day 6, just prior to death. Necrosis was noted in association with neutrophil aggregates in six of eight control animals at days 1–6, whereas necrosis was rarely seen in CpG treated animals (one of eight animals at days 1–6).

**Table 1:** Summary of lesions observed and their characteristics.^a^

Treatment	Day post infection	Average # of lesions per section	Lesion size range
CpG	1	0.3	4 to 10 alveoli
CpG	2	0.8	2 to 18 alveoli
CpG	3	4.3	2 to 20 alveoli
CpG	6	0.3	0 to 1 alveolus
CpG	13	0.0	No lesions seen
CpG	15	1.0	0 to 2 alveoli
CpG	25	1.0	1 to 2 alveoli
Control	1	1.1	5 to 25 alveoli
Control	2	12.5	2 to 30 alveoli
Control	3	3.8	1 to 40 alveoli
Control	6	2.00	1 to 40 alveoli

a Lung samples from two animals from each group and time point were examined after routine H&E staining. Lesion count is estimated as the average number of lesions per lung section for the two animals. Lesion size is reported as the number of alveoli filled by neutrophils.

### Effect of CpG ODN on recruitment of inflammatory cell populations

**Figure 4:**
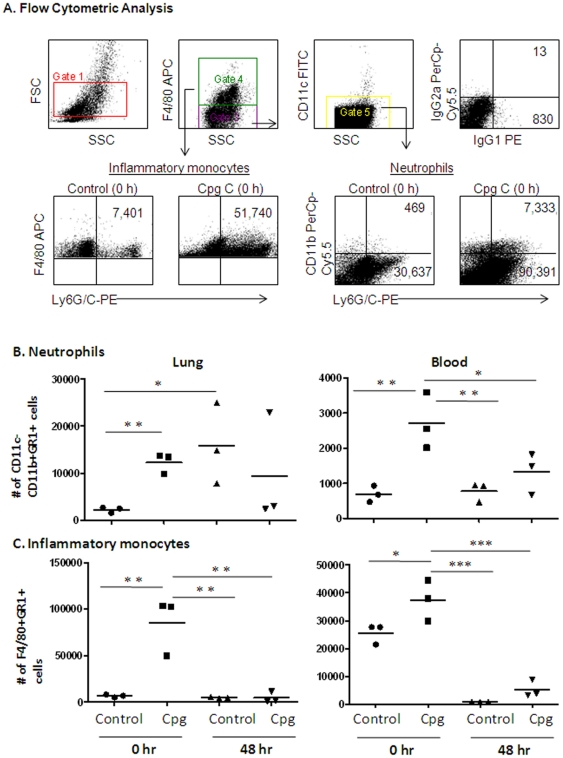
Flow cytometry analysis of inflammatory cell populations. Cells from blood and lungs were isolated at time 0 and 48 h after CpG ODN administration, but before infection, and 48 h post infection. Cells were labeled with monoclonal antibodies Ly6G/C-PE, CD11c-FITC, CD11b-PerCpCy5.5, F4/80-APC with corresponding isotype controls and analyzed using FCS Express Version 3. Shown in (A) is the flow cytometric analysis of leukocytes, side scatter and forward scatter characteristics of isolated cells (Gate 1), gating strategy for analysis of F4/80+ (monocyte/macrophages, Gate 2) and F4/80− (Gate 3) and CD11c+ (DC, Gate 4) cell populations. Shown are representative plots for numbers of inflammatory monocytes (F4/80+Ly6G/C+ cells) and neutrophils (F4/80−CD11c−CD11b+Ly6G/C+ cells) following CpG treatment and *B. pseudomallei* infection. Results are representative of 3 animals per group. Shown in B and C are a summary of monocyte and neutrophil numbers in lung and blood of CpG and non-treated animals prior to and following infection. Data are presented as mean ± SEM using one-way ANOVA followed by a Dunnett's multiple comparison test for group comparisons (GraphPad Software v4.0). Statistically significant values are designated as follows: *, p<0.05; **, p<0.01.

In a separate study, groups of mice were either mock (PBS) or CpG treated (i.n.) and sacrificed at time 0 (time of infection and 48 h after CpG treatment), and 48 h post infection with 2LD_50_ of *B. pseudomallei*. Peripheral blood and lung leukocytes were isolated and flow cytometric analysis was performed to quantitate inflammatory cell populations. Assessment was done based on number of cells in equal volume of sample (blood, total lung homogenate) from each animal. The cell populations were identified based on surface phenotype markers; F4/80^+^Gr1^+^ (inflammatory monocytes), F4/80^−^CD11c^−^CD11b^+^Gr1^+^ (neutrophils) and F4/80^+^Gr1^−^ (macrophages) (Figure 4A). We observed a robust increase in inflammatory monocytes and neutrophils in both the blood and lungs by 48 hours after CpG administration and prior to infection (Figure 4B and C). Animals which received 20 µg CpG ODN intranasally had 1.5 times higher level of inflammatory monocytes in blood and almost 13 times higher level of inflammatory monocytes in lungs prior to infection (time 0) as compared to control mice. Similarly, CpG ODN treatment resulted in significantly increased numbers of neutrophils in the blood and lungs (Figure 4B and C). The numbers of neutrophils observed 48 h after CpG administration, but prior to infection, was 3.9 times higher in blood and 5.8 times higher in lungs as compared to controls. These significant differences in numbers of inflammatory cell populations in blood and lungs between CpG treated and not treated groups were no longer observed by 48 h post infection. In contrast, neutrophils were abundant in the lung of both CpG- and non-treated groups by 48 h post-infection while monocyte numbers were not different from control animal baseline values (Figure 4B and C). Our results indicate that early activation of innate immunity by CpG ODN allowed for effective control of this invading pathogen at the site of infection and contributed to the survival of CpG treated animals.

### Effect of CpG ODN on chemokine/cytokine profile

**Figure 5:**
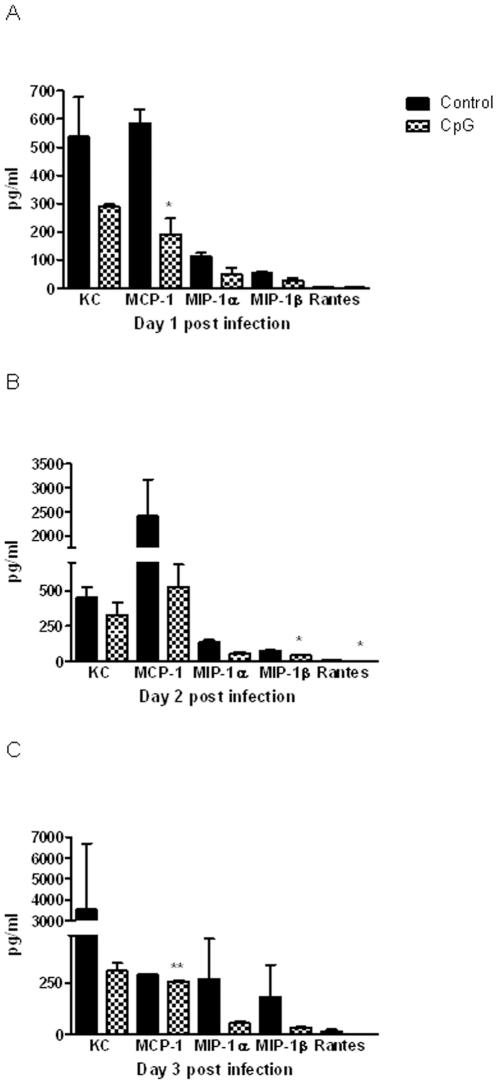
Murine chemokines responses. Level of chemokines in plasma in pg/ml of control and CpG treated animals during first three days post-infection (Panels: A- day 1; B- day 2; C- day 3). Plasma from two mice was analyzed for each time point. Data are presented as mean ± SEM *p<0.05 ***p<0.001.

**Figure 6:**
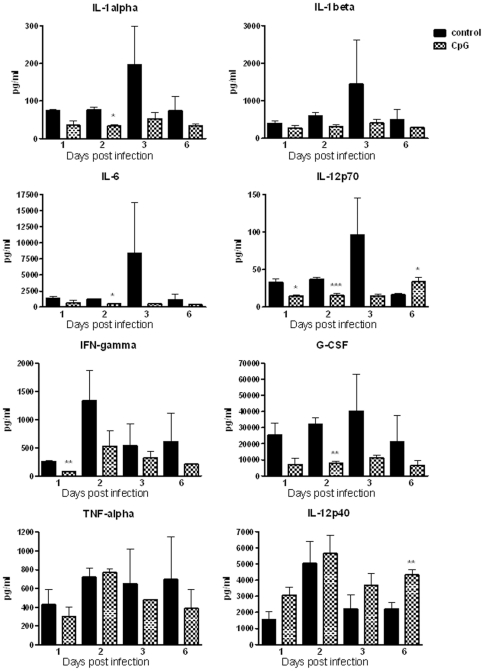
Murine cytokine responses. Level of individual cytokines in plasma (pg/ml) of control and CpG treated animals during the first six days post-infection. Blood from two mice was analyzed for each time point. Data are presented as mean ± SEM *p<0.05 ***p<0.001.

**Figure 7:**
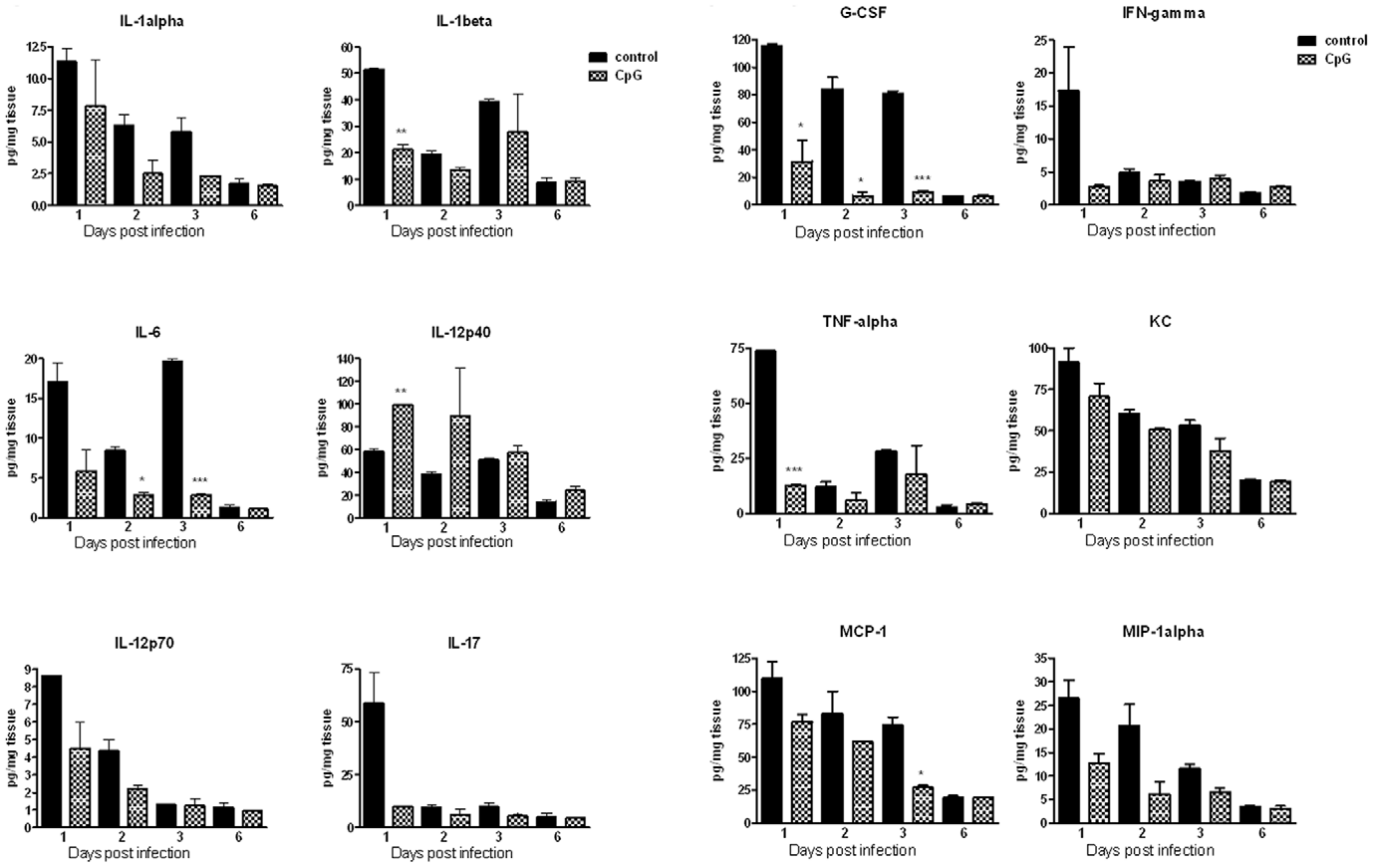
Cytokines and chemokines from infected organs. Level of individual cytokines and chemokines (pg/mg tissue) in lungs of control and CpG treated animals during the first six days post-infection. Two mice were sacrificed from each group at each time point. Lungs were homogenized in PBS and cytokine and chemokine analysis was performed using Bio-Plex cytokine assay (Panels A and B). Data are presented as mean ± SEM *p<0.05 ***p<0.001.

To determine the immune responses associated with protection generated by CpG administration against the acute form of disease caused by *B. pseudomallei*, we evaluated chemokines/cytokines produced post infection in blood, lung and spleen. The cytokine response in the first three days post infection corresponded with detrimental disease progression as most control animals died between days 3 and 6. Without the CpG pretreatment, *B. pseudomallei* infection was associated with robust, increased levels of chemokines and cytokines in blood, lungs and spleens, peaking within three days post infection (Figures 5, 6, and 7, Figure S1). All measured chemokines were strongly up-regulated in the plasma of control animals, with the amount of MCP-1 at days 1 and 3, MIP-1β and RANTES at day 2, being statistically different as compared to that of CpG treated mice (Figure 5). Starting at day 1 post infection, control animals also had higher levels of several cytokines, including IL-1α, IL-1β, IL-6, IL-12p70, and IFN-γ in plasma, additionally IL-17 and TNF-α and chemokines (KC, MCP-1, MIP-1α) were elevated in lungs (Figures 6 and 7). G-CSF was the most abundant cytokine produced in plasma, lungs and spleens of control animals throughout the first 3 days post infection with consistent statistical differences in lungs between control and CpG treated mice (Figures 6 and 7, Figure S1). While the level of G-CSF was abundant in control animals, the IL-12p40 was the only cytokine consistently elevated in CpG treated mice as compared to controls. No significant differences due to treatment were noted for several other molecules (IL-2, IL-3, IL-4, IL-5, IL-9, IL-10, IL-13, GM-CSF, MIP-1β, or RANTES) measured via the 23-plex platform. Statistical differences were detected only on day 1 post infection in lungs and in plasma at day 6. The cytokines previously shown to be important in protection against *B. pseudomallei*, such as TNF-α, IFN-γ and IL-12p70, were detected at moderate levels in CpG treated BALB/c mice throughout the course of infection (Figures 6 and 7). Most of cytokines and chemokines measured in the lungs were detected in much higher level in control animals. We observed a strong induction (burst) of cytokines and chemokines in lungs of control animals at day 1 with declining trend during the next three days, although the quantities were still higher when compared to CpG treated animals. The significant differences were seen in IL-1β (day 1), IL-6 (days 2 and 3), G-CSF (days 1, 2 and 3), TNF-α (day 1) and MCP-1 (day 3). By day 6, production of cytokines dropped significantly and was similar in both groups.

## Discussion

The pathogenesis of melioidosis and the accompanying host immune response varies widely based on host susceptibility and route of delivery. Studies of *Burkholderia* infection in murine models have demonstrated a distinct susceptibility of BALB/c mice compared to C57BL/6 mice [25], [26]. Because BALB/c mice are very susceptible to both intravenous and intranasal *B. pseudomallei* infections, this mouse strain was chosen to investigate in detail the innate immune responses generated by unmethylated CpG oligodeoxynucleotides. The CpG ODNs are known to be potent immunomodulators and induce highly effective protective immunity in a number of infectious diseases including tularemia [6], listeriosis [27], *Leishmania* [28] and *B. mallei* or *B. pseudomallei* infections [4], [5], [6], [9], [15]. However, the positive immunostimulatory effect of CpG can be diminished in the case of infection with highly virulent bacterial strains which actively impair the host immune response such as *Francisella tularensis* Schu S4 strain [3]. Consistent with these observations, we showed that pretreatment with CpG ODN types B or C administered i.n. significantly prolonged the survival of *B. pseudomallei* infected BALB/c mice. The survival was dependent on infectious dose and varied from 100% at 2 LD_50_ to 50% with 4 LD_50_ (Figure 1). In all experiments, day 3 post infection was shown to be a critical point and most deaths in the control group occurred within days 3 to 6 post infection. Our data suggested that in the case of acute pulmonary melioidosis, the initial responses generated by innate immunity are extremely important for protection.

The kinetics of bacterial clearance or colonization in lungs and spleens were determined at 6 different time points after challenge to investigate contributing factors to protection generated by CpG ODN type C. Control BALB/c mice had an overwhelming bacterial load in both the lungs and spleen (Figure 2). Previous studies showed that BALB/c mice infected intravenously with *B. pseudomallei* developed elevated bacterial loads in the lung and spleen with considerably fewer bacteria in the liver [25]. The difference in bacterial numbers between control and CpG treated animals in our studies was observed from day 1 and continued until day 6 post infection, when most control animals died. Our observations are in agreement with experiments with BALB/c mice pretreated with CpG ODN, type B and i.p. infected with *B. pseudomallei* [5]. Bacterial levels detected in blood were low and found in small proportion of CpG pre-treated mice. CpG, type C pre-treated mice in our study maintained low level of bacteria in lungs with an increased observed only at day 3 post infection. After this time point, bacteria in CpG pre-treated mice were well controlled in lungs till end of the study. The organism was also detected in low numbers very early (day 1 post infection) in the spleen in all animals indicating the early spread of bacteria into the blood stream. Colonization of the bacteria in the spleen observed during late course of infection indicate that animals did not develop efficient clearance mechanism in that organ and bacteria were able to grow rapidly later, reaching elevated numbers at the end of the study (day 21). In support of the greater survival that we and others have observed, here it was demonstrated that CpG pre-treatment of BALB/c mice led to recruitment of innate immune cell populations that promotes clearance of the bacteria, at the primary site of infection (lung). The non-activated state of the innate immune system in the control animals likely allowed the rapid growth of *B. pseudomallei* in lungs, leading to a subsequent robust activation of pro-inflammatory responses which were not adequate for clearance of the bacteria and yet had a negative outcome.

Control of bacterial growth in our studies correlated with the early recruitment of large numbers of inflammatory monocytes and neutrophils to the site of infection (lungs) in CpG treated animals. We found a significantly higher number of inflammatory monocytes and neutrophils in blood and lungs in animals pretreated with CpG ODN prior to infection. Neutrophils and monocytes are a key component of the innate immunity and are important for protection against pathogens. They have a critical early role in resistance, both by direct antimicrobial activity (release of antibacterial molecules and proteinases) and by production of immunoregulatory chemokines and cytokines that recruit and activate other immune cells. Early recruitment of monocytes and neutrophil populations in CpG treated animals would very likely contribute to control of bacterial growth and protection. Neutrophils have been shown to play critical roles in resistance to *B. pseudomallei* infection [29]. They displayed potential microbicidal capacity by production of reactive oxygen intermediates and increased early pro-inflammatory cytokine production. We detected elevated levels of G-CSF, IL-6, and KC, which supported enhancement of migration, activation and proliferation of neutrophils. Our study showed that in the case of a fast progressing disease, such as melioidosis, the time of activation of host defense is a very important factor in successful treatment. The early activation of innate immunity and pro-inflammatory responses were critical to successful eradication of the pathogen, whereas elevated cytokine levels at later time points was likely the result of poorly controlled bacterial growth. Our results are in agreement with a model of pro-inflammatory responses of innate immunity proposed by Netea et al., [20]. The authors emphasized that in acute severe infection, the relative deficiencies in innate immunity could lead to rapid multiplication of the invading pathogen with a massive secondary reaction of the host which could ultimately lead to shock and death.

It has been shown previously that *B. pseudomallei* infection in BALB/c mice promoted a strong local inflammatory response, but an increased level of cytokines and chemokines was not always effective in reducing the bacterial load [5], [24], [25]. The responses did not protect the animals but likely contributed to pathology through tissue destruction [5], [24]. Comprehensive analysis of cytokine and chemokine profiles after *B. pseudomallei* infection in our study showed that moderate production of cytokines and chemokines during the early stage of infection in mice treated with CpG ODN was associated with greater protection against *B. pseudomallei*. This is in agreement with previous findings indicating that protection against acute septicemic melioidosis in BALB/c mice immunized with CpG ODN type B was associated with a reduction of bacterial load and moderate production of pro-inflammatory cytokines [5]. The authors of that study analyzed cytokine level only in serum after i.p. challenge with *B. pseudomallei* strain H1038. The cytokine analysis was limited to a few cytokines (IFN-γ, TNF-α, and IL-12p70) also shown to be very important in innate host resistance to the closely related bacterium *B. mallei* [30]. Wongratanacheewin *et al.*, measured the cytokine level in the animals prior to infection and found small but highly significant early increases in the level of IL-12p70 and IFN-γ, 24 h after CpG ODN injection [5]. This is a very important finding because these two cytokines are critical in stimulating activation of macrophages, NK cells and other innate immunity cells. In mice infected with *B. pseudomallei*, NK cells were shown as the principle source of IFN-γ while monocytes were the primary source of TNF-α [29]. Although our study indicates that a large production of these cytokines was not necessarily beneficial for either bacterial burden or survival of the control mice, the time of very early release of these cytokines seems to be a very important factor contributing to protection.

In a separate study, it was shown that CpG ODN, type B enhanced phagocytosis of *B. pseudomallei* and induced nitric oxide synthase and nitric oxide production by murine macrophages *in vitro* [31], [32]. Here, we demonstrated that protected CpG pre-treated mice had a moderate level of IL-12p70 but a much higher level of IL-12p40. The importance of IL-12 for offering limited protection against the related pathogen *Burkholderia mallei* has been shown in BALB/c mice [33]. Another study of pulmonary *B. mallei* infection has shown that recruitment of inflammatory monocytes and DC to the lungs and local production of IL-12 were important initial responses required for early protection. [34]. It is possible that upon early activation of TLR9 by CpG, the resident or recruited antigen presenting cells are able to produce a greater amount IL-12p40. The role of p40 is not fully understood, and a recent study presented evidence that this molecule activates pro-inflammatory responses in experimental allergic encephalomyelitis [35]. Another potential explanation for the difference in IL-12 expression between the groups is negative regulation by IL-6. As IL-12 is negatively regulated by IL-6 [36], the higher level of IL-6 in the non-treated group may suppress IL-12 activation following infection in this group.

Histopathological examination of lung sections demonstrated microabscesses in infected lungs, mimicking human respiratory melioidosis [37]. Of note, at 24 h and 48 h, neutrophil aggregates were larger and more numerous in the control animals compared to the CpG treated animals. As expected, H&E staining of lung tissue demonstrated large clusters of neutrophils with stronger signs of inflammation and lesions in control animals. Interestingly, histological analysis of tissue from patients with melioidosis presented predominantly neutrophilic lesions at infected sites [38]. Increased infiltration of neutrophils occurs in many acute lung diseases and it is part of lung's defense system but has also been implicated in pathogenic processes. Despite the fact that we detected early infiltration of lungs with large number of inflammatory monocytes and neutrophils in CpG treated animals, the lung of these animals were cleared from pathogen without causing extensive lung damage seen in control animals. This observation suggests that in CpG treated mice, clearance of apoptotic neutrophils by professional phagocytic cells may be properly initiated early during infection. A study showing that CpG ODN enhances ingestion of apoptotic neutrophils by macrophages *in vitro* supports this observation [16].

In summary, CpG pretreatment of susceptible BALB/c mice results in recruitment of neutrophils and monocytes to the lung, better control of bacterial growth and survival, simultaneously with an early, moderate, production of pro-inflammatory cytokines and chemokines. Our study indicates that during infection, a large production of these cytokines was not necessarily beneficial for either bacterial burden or survival of mice. In the absence of a vaccine or effective antibiotic therapies, the beneficial immunomodulatory effect of CpG ODN could offer an alternative prophylactic approach to protect populations at risk of exposure to the weaponized form of the bacteria.

## Methods

### Ethics Statement

This study was carried out in strict accordance with the recommendations in the Guide for the Care and Use of Laboratory Animals of the National Institutes of Health. The protocol was approved by the Animal Care and Use Committee of the University of Texas Medical Branch (Protocol Number: 0503014A).

### Bacterial strain

*B. pseudomallei* strain K96243 was cultured on Luria-Bertani supplemented with 4% glycerol (LBG) agar plates for 48 h at 37°C. Isolated colonies were sub-cultured to LBG broth, and cultures were incubated at 37°C until optical density readings at 600 nm (OD_600_) reached an exponential phase of growth. Bacteria were pelleted by centrifugation, washed and re-suspended in sterile 1X phosphate-buffered saline (PBS, pH 7.4) to obtain the desired CFU/ml. All procedures were performed in a biosafety level 3 laboratory.

### Mice

Female, 8- to 10-week-old, BALB/c mice were obtained from Harlan Sprague Dawley, Inc. (Indianapolis, Indiana). Animals were provided with rodent feed and water *ad libitum* and maintained on 12 h light cycle. All animal experiments were performed under animal biohazard containment level 3 conditions.

### CpG ODNs administration and *B. pseudomallei* challenge

Groups of 10 or 20 animals were inoculated via the intranasal (i.n.) route with dose equal to 2–4 LD_50_ of *B. pseudomallei*, strain K96243 depending on experiments, in a total volume of 50 µl in PBS solution divided into both nares. The 1 LD_50_ dose was established in previous studies as 3.12×10^2^ for intranasal route in BALB/c mice. Phosphorothioate-stabilized CpG oligodeoxynucleotides were purchased from Coley Pharmaceutical Group, Ottawa, Canada. CpG ODN type B 1826 (TCC ATG ACG TTC CTG ACG TT) or type C 2395 (TCG TCG TTT TCG GCG CGC GCC G) was administered 48 h prior challenge via i.n. route, 20 µg per mouse in 50 µl of water into both nares (25 µl each). The post-challenge administration of CpG ODN, type C (1 h post-challenge) was also tested. Control animals received corresponding non-CpG ODN 2138 for type B (TCC ATG AGC TTC CTG AGC TT) or 2137 for type C (TGC TGC TTT TGT GCT TTT GTG CTT) respectively in the same amount, route and time.

### Bacterial load determination

Two animals from each group (control, not treated and CpG treated) were sacrificed on days 1, 2, 3, 6, 13, and at the end of the study, blood, lungs and spleens were harvested for CFU determination, cytokines and chemokines analysis or histological examinations. Organs were weighed, half of each organ was homogenized in 1 ml sterile PBS, and serial 10-fold dilutions were plated in duplicates on LBG agar plates and incubated at 37°C for 2 days prior to CFU determinations. The CFUs were expressed as the mean ± SEM. Remaining homogenates were spun in a microcentrifuge and stored frozen for cytokine analysis. Bacterial load determination was only performed in experiments testing prophylactic administration of CpG ODN, type C.

### Histopathology of the lungs

Two animals from each group (control, not treated and CpG treated) were sacrificed on days 1, 2, 3, 6, 13, and at the end of the study. Lungs were instilled with formalin, processed and paraffin-embedded. Hematoxylin and Eosin stained slides, containing 1–6 sections of lung, were examined by a pathologist (Dr. Aronson) for lesion number and lesion size. The average number of lesions per section was determined for each animal. Lesion size was estimated in relation to the number of alveoli involved. The presence or absence of necrosis associated with neutrophil aggregates was noted.

### Cell isolation and flow cytometry

Blood (200 µl) was collected by cardiac puncture and lungs were harvested from 8-week old female BALB/c mice (Harlan Laboratories) following sacrifice as approved by the Institutional Animal Care and Use Committee. Single cell suspensions of lung tissue were prepared from harvested lungs following tissue disruption as previously described [39]. Red blood cells (RBC) in lung and peripheral blood samples were lysed using RBC Lysis Buffer (Sigma) according to the protocol provided by the manufacturer. A white blood cell count and analysis of cell viability was performed by trypan blue exclusion. Samples were labeled with fluorescent monoclonal antibodies for flow cytometric analysis, using procedures that we have previously described [40]. The following monoclonal antibodies (mAb) against mouse antigens were purchased from BD Biosciences: Ly6G/C-PE, CD11c-FITC, CD11b-PerCpCy5.5, and F4/80-APC. Corresponding isotype controls were also purchased from BD Biosciences: IgG2b-PE, IgG2b-FITC, IgG2a-APC, or IgG1-PerCPCy5.5. Cells were then washed and re-suspended in 500 µl of 4% ultrapure formaldehyde (Polysciences Inc.). Samples were fixed for 48 h, with fresh 4% formaldehyde replacement at 24 h, and sterility confirmed by lack of growth of *B. pseudomallei* on selective growth agar plates. A total of 400 µl of sample (blood or lung suspension) was acquired on a FACS Canto (BD Biosciences, UTMB Flow Cytometry and Cell Sorting Core Facility) and compensation for spectral overlap was performed using FACS DIVA software (BD Biosciences). Isotype- and fluorochrome-matched non-specific control antibodies were used to determine background fluorescence. Analysis was performed using FCS Express version 3 (De Novo Software) as we have previously described [40]. Data is presented as the number of gated events corresponding to the expected live leukocyte side scatter and forward scatter gate. Assessment is based on the number of F4/80^+^Gr1^−^ (macrophages), F4/80^+^Gr1^+^ (inflammatory monocytes), and F4/80^−^CD11c^−^CD11b^+^Gr1^+^ (neutrophils) cells in the live leukocyte gate. Data are shown as mean ± SEM. One-way ANOVA followed by a Dunnett's multiple comparison test for group comparisons (GraphPad Software v4.0). Statistically significant values are designated as follows: *, p<0.05; **, p<0.01.

### Measurement of blood, lung and spleen cytokines and chemokines

Bio-Plex cytokine assays are optimized for the Bio-Plex system, which uses xMAP detection technology. Multiplex premixed panels of 23 mouse cytokines/chemokines was detected in mouse samples (IL-1α, IL-1β, IL-2, IL-3, IL-4, IL-5, IL-6, IL-9, IL-10, IL-12 (p40), IL-12 (p70), IL-13, IL-17, EOTAXIN, G-CSF, GM-CSF, IFN-γ, KC, MCP-1, MIP-1α, MIP-1β TNF-α, RANTES). A detailed cytokine or chemokine analysis was only performed in experiments testing prophylactic administration of CpG ODN, type C.

### Statistical analysis

Survival curves were calculated by Kaplan Meier survival analysis with log-rank tests between groups using GraphPad Prism (v.4.03 for Windows). All other data were analyzed by Student's t-test. P value≤0.05 was considered significant.

## Supporting Information

Figure S1 **Cytokines and chemokines from infected spleens.** Level of individual cytokines and chemokines (pg/mg tissue) in spleens of control and CpG treated animals during the first six days post-infection. Two mice were sacrificed from each group at each time point. Spleens were homogenized in PBS and cytokine and chemokine analysis was performed using Bio-Plex cytokine assay. Data are presented as mean ± SEM *p<0.05 ***p<0.001.
(TIF)
