# Supplementary figures and images for: Prophylactic Application of CpG Oligonucleotides Augments the Early Host Response and Confers Protection in Acute Melioidosis
Source: PLoS One. 2012 Mar 20;7(3):e34176. doi: 10.1371/journal.pone.0034176 (PMC3309019; doi:10.1371/journal.pone.0034176)

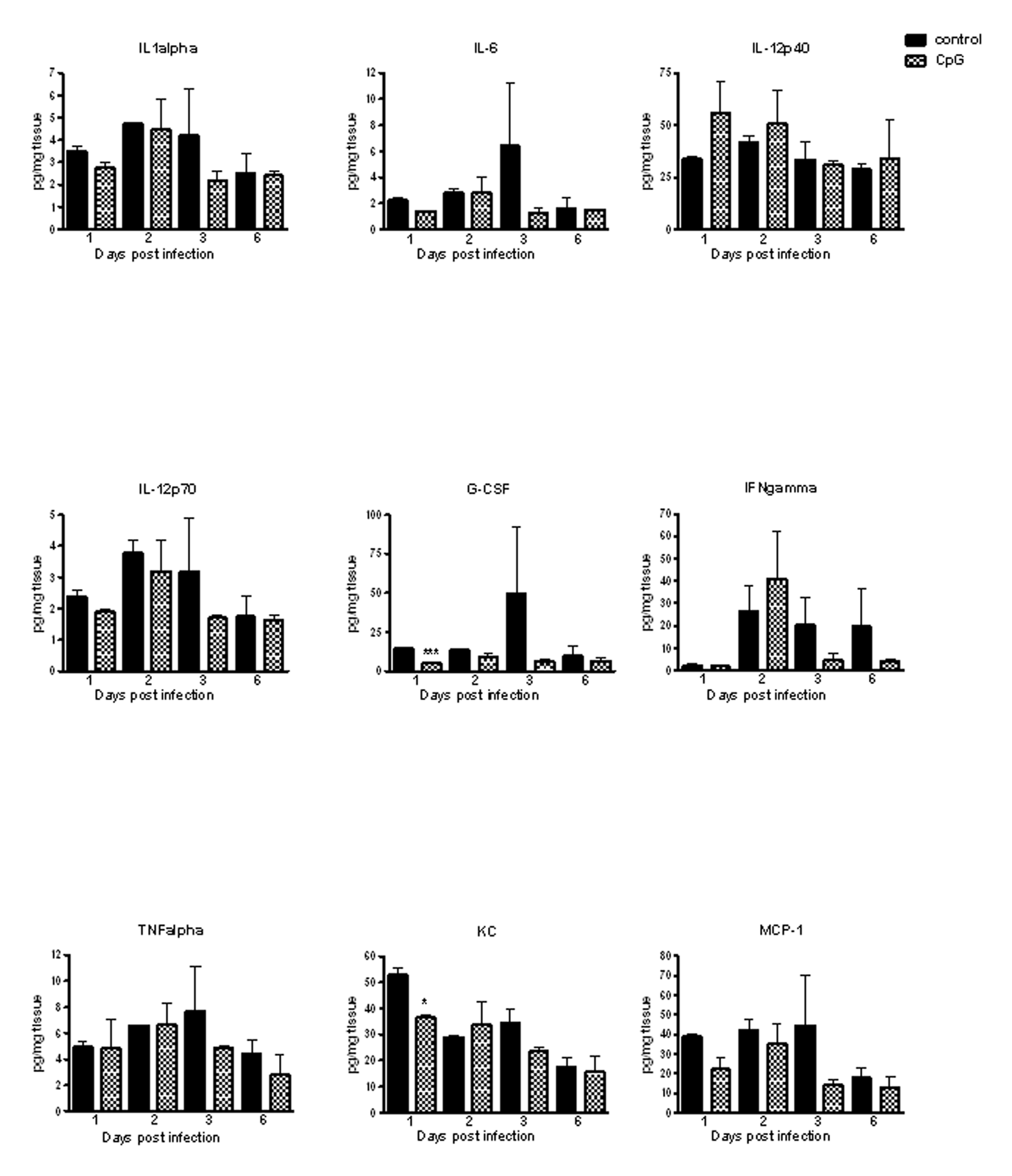

Supplement: Figure S1 — Cytokines and chemokines from infected spleens. Level of individual cytokines and chemokines (pg/mg tissue) in spleens of control and CpG treated animals during the first six days post-infection. Two mice were sacrificed from each group at each time point. Spleens were homogenized in PBS and cytokine and chemokine analysis was performed using Bio-Plex cytokine assay. Data are presented as mean ± SEM *p<0.05 ***p<0.001. (TIF) [file pone.0034176.s001.tif]
